# Understanding the mechanisms of fatigue in multiple sclerosis: linking interoception, metacognition and white matter dysconnectivity

**DOI:** 10.1093/braincomms/fcae292

**Published:** 2024-09-11

**Authors:** Iulia Danciut, Charlotte L Rae, Waqar Rashid, James Scott, Marco Bozzali, Mihaela Iancu, Sarah N Garfinkel, Samira Bouyagoub, Nicholas G Dowell, Dawn Langdon, Mara Cercignani

**Affiliations:** Clinical Imaging Sciences Centre, Department of Clinical Neuroscience, Brighton and Sussex Medical School, University of Sussex, Falmer, Brighton BN1 9RR, UK; Department of Neurology, Hull Royal Infirmary, Hull HU3 2JZ, UK; School of Psychology, University of Sussex, Falmer, Brighton BN1 9QH, UK; Department of Neurology, St George’s Teaching Hospitals, London SW17 0QT, UK; Psychosis Studies, Institute of Psychiatry, Psychology and Neuroscience, King’s College London, London SE5 8AB, UK; ‘Rita Levi Montalcini’ Department of Neuroscience, University of Torino, 10126 Turin, Italy; Department of Medical Informatics and Biostatistics, Faculty of Medicine, ‘Iuliu Haţieganu' University of Medicine and Pharmacy, 400349 Cluj-Napoca, Romania; Institute of Cognitive Neuroscience, UCL, Queen Square, London WC1N 3AZ, UK; Clinical Imaging Sciences Centre, Department of Clinical Neuroscience, Brighton and Sussex Medical School, University of Sussex, Falmer, Brighton BN1 9RR, UK; Clinical Imaging Sciences Centre, Department of Clinical Neuroscience, Brighton and Sussex Medical School, University of Sussex, Falmer, Brighton BN1 9RR, UK; Psychology Department, Royal Holloway University of London, Egham, Surrey TW20 0EX, UK; Clinical Imaging Sciences Centre, Department of Clinical Neuroscience, Brighton and Sussex Medical School, University of Sussex, Falmer, Brighton BN1 9RR, UK; Cardiff University Brain Research Imaging Centre, School of Psychology, Cardiff University, Cardiff CF24 4HQ, UK

**Keywords:** multiple sclerosis, cognitive fatigue, white matter, interoceptive insight, diffusion MRI

## Abstract

One of the most prominent symptoms in multiple sclerosis is pathological fatigue, often described by sufferers as one of the most debilitating symptoms, affecting quality of life and employment. However, the mechanisms of both, physical and cognitive fatigue in multiple sclerosis remain elusive. Here, we use behavioural tasks and quantitative MRI to investigate the neural correlates of interoception (the ability to sense internal bodily signals) and metacognition (the ability of the brain to assess its own performance), in modulating cognitive fatigue. Assuming that structural damage caused by multiple sclerosis pathology might impair the neural pathways subtending interoception and/or metacognition, we considered three alternative hypotheses to explain fatigue as a consequence of, respectively: (i) reduced interoceptive accuracy, (ii) reduced interoceptive insight or (iii) reduced global metacognition. We then explored associations between these behavioural measures and white matter microstructure, assessed by diffusion and magnetisation transfer MRI. Seventy-one relapsing-remitting multiple sclerosis patients participated in this cross-sectional study (mean age 43, 62% female). Patient outcomes relevant for fatigue were measured, including disability, disease duration, depression, anxiety, sleepiness, cognitive function, disease modifying treatment and quality of life. Interoceptive and metacognitive parameters were measured using heartbeat tracking and discrimination tasks, and metacognitive visual and memory tasks. MRI was performed in 69 participants, including diffusion tensor MRI, neurite orientation dispersion and density imaging and quantitative magnetisation transfer. Associations between interoception and metacognition and the odds of high cognitive fatigue were tested by unconditional binomial logistic regression. The odds of cognitive fatigue were higher in the people with low interoceptive insight (*P* = 0.03), while no significant relationships were found between fatigue and other interoceptive or metacognitive parameters, suggesting a specific impairment in interoceptive metacognition, rather than interoception generally, or metacognition generally. Diffusion MRI-derived fractional anisotropy and neurite density index showed significant (*P* < 0.05) negative associations with cognitive fatigue in a widespread bilateral white matter network. Moreover, there was a significant (*P* < 0.05) interaction between cognitive fatigue and interoceptive insight, suggesting that the poorer the white matter structure, the lower the interoceptive insight, and the worse the fatigue. The results point towards metacognitive impairment confined to the interoceptive domain, in relapsing-remitting patients with cognitive fatigue. The neural basis of this impairment is supported by a widespread white matter network in which loss of neurite density plays a role.

## Teaser

In multiple sclerosis, fatigue impacts daily life. Danciut *et al.* investigated how internal bodily signals and self-assessment affect cognitive fatigue. In 71 patients with multiple sclerosis, those with lower insight into bodily signals experienced higher cognitive fatigue. MRI scans revealed microstructural damage in areas linked to bodily signals perception.

## Graphical Abstract

**Graphical Abstract:**
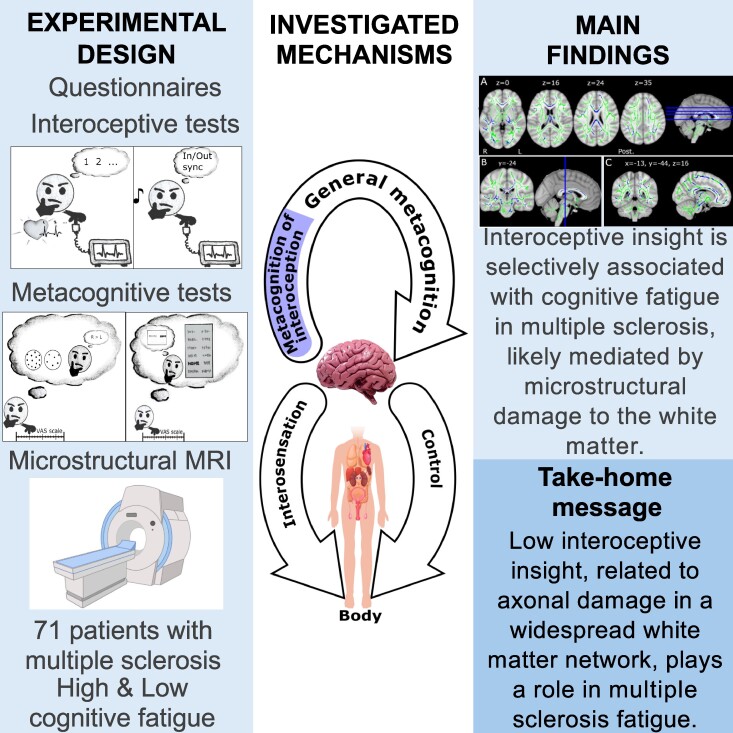

See A. Chalah and S. Ayache (https://doi.org/10.1093/braincomms/fcae302) for a scientific commentary on this article.

## Introduction

Fatigue is among the most common symptoms in multiple sclerosis (MS), with significant impact on quality of life.^1,2^ It affects up to 80% of people with MS^3^ and tends to persist over time.^4^ It can be present early in the disease course,^5^ and in the absence of any other MS symptom,^6^ or even precede other symptoms by years.^7,8^ Furthermore, fatigue contributes to the economic burden of MS,^9^ is implicated in almost all work difficulties in MS,^9^ and affects both productivity loss and employment status.^10^ People with MS experience physical, cognitive and psychosocial fatigue.^11^ Psychosocial fatigue encompasses emotional and social aspects of fatigue, including feelings of sadness, irritability and social withdrawal. Cognitive fatigue is described as the inability to sustain cognitive task performance due to mental exhaustion^12^ and can be measured using a continuous information processing speed task.^13,14^ However, this assessment better suits the concept of fatiguability—a ‘state’ fatigue^15^ rather than a ‘trait’ fatigue. In general, ‘fatiguability’ refers to the propensity or susceptibility to become fatigued in response to physical or cognitive exertion, and objective measures can be used for it, i.e. by observing and quantifying a decrease in performance during a fatiguing task.^16^ On the other hand, ‘fatigue’ refers to the more subjective feeling of tiredness, exhaustion or lack of energy that can affect physical, cognitive and psychosocial functioning. Changes in objective fatigability may not go in parallel with subjective feelings of impairment, for which objective measures are scarce, and relying on the use of questionnaires.^17^

The underlying mechanisms of fatigue in MS are not fully understood. Inflammation is most likely involved, possibly through a combination of processes.^18^ The release of pro- and anti-inflammatory cytokines could lead to a cascade of events at both central and peripheral level.^19^ One of the putative mechanism is through monoaminergic signalling, and particularly the synthesis of dopamine.^20^ Fatigue would result from a mismatch between the perceived task-related effort and benefit, which has also been attributed to an abnormality in reward processing within the cortico-striatal pathways. More recently, it has been observed that individuals experiencing high levels of MS fatigue exhibit diminished connectivity between key areas of the brain noradrenaline circuits when compared to those with lower levels of fatigue.^21^ Cytokines, however, may also interfere with the hypothalamus–pituitary axis (HPA) activation and anti-inflammatory cholinergic pathways.^22^ They can activate both central and peripheral immune processes.^23^ This communication between the immune system and the brain primarily occurs through vagal afferents, which are activated by proinflammatory mediators. These signals are then relayed to the ventromedial posterior thalamus and mid-insular cortex. This interoceptive pathway^24^ plays a critical role in linking immune responses to the brain during sickness behaviour and is also important in understanding fatigue.^25,26^ Through this pathway, peripheral immune processes can influence the activation of the HPA axis, thus connecting immunological and endocrinopathic theories of fatigue.^24,27^ As MS causes widespread damage to brain tissue, it is conceivable that communication between the components of this network might be impaired, resulting in deficits in one or more of the interoceptive domains.^28^ Consistently, neuroimaging studies support the hypothesis that MS fatigue may involve a complex neural network. Overall, structural,^29^ functional^30^ and connectivity^31^ findings point at the involvement of a cortico-striato-thalamo-cortical loop.^32^

Interoception is formally defined as the process by which the nervous system senses, interprets and integrates signals originating from within the body, providing a moment-by-moment mapping of the body’s internal landscape across conscious and unconscious levels.^33^ Interoception encompasses a number of distinct and interrelated bodily axes^22^; however a large proportion of empirical interoceptive research to date has focused on cardiac interoception, as heartbeats are discrete and easily quantifiable events. Interoception can be delineated across different hierarchical levels, including the neural processing of afferent signals to higher order measures pertaining to the attention and interpretation of internal bodily signals.^34^ A central tenet of interoception is ‘interoceptive accuracy’, defined as the accuracy with which interoceptive afferent signals, such as heartbeats, can be detected. Self-report measures of interoception, such as questionnaires assessing ‘awareness’ into interoceptive signals do not necessarily align with interoceptive accuracy.^35^ A metacognitive measure of interoception, previously termed ‘interoceptive awareness’,^35^ and now referred to as ‘interoceptive insight’,^33^ assesses whether people have good insight into their interoceptive abilities, e.g. does participants’ confidence correlate with their performance accuracy. Finally, self-reported ‘awareness’ into interoception has been conceptualized as a measure of ‘interoceptive sensibility’.

Impairment in either interoceptive accuracy or interoceptive insight have been proposed to explain fatigue in MS, within the framework of the allostatic self-efficacy (ASE) theory.^18,28,36^ In addition to proposals that fatigue can be explained by a specifically interoceptive metacognitive dysfunction (induced by chronic dyshomeostasis), it is possible that fatigue is caused by a more general metacognitive impairment. In the context of MS, tissue damage to both white and grey matter, combined with inflammation, might lead to maladaptive network recruitment, resulting in altered brain-body communication and/or metacognition of interception.^18^ A recent study investigated these hypotheses directly, using self-reported measures of interoceptive insight based on questionnaires, and found that fatigue in MS is associated with interoceptive insight, but not with exteroceptive metacognition or autonomic dysfunction.^37^

In this paper, we independently replicate and complement these findings by investigating the role of experimentally measured interoception accuracy, interoceptive insight and exteroceptive metacognition in fatigue. Furthermore, building upon the hypothesis that any impairment to these processes might result from a loss of connectivity due to MS-related brain tissue abnormalities, we link these behavioural outcomes to microstructural white matter biomarkers derived from neuroimaging. Although both focal demyelinating lesions and diffuse tissue damage can lead to loss of connectivity between segregated areas of the brain, the majority of studies investigating the relationship between T2 lesion load and fatigue found that lesion load was not related to the severity of the fatigue^38-40^—suggesting that microstructural damage might be more relevant in the context of fatigue. At the microstructural level, MS pathology can affect axons, glial cells and myelin, all of which may impair connectivity. We used two complementary MRI techniques, namely diffusion and magnetisation transfer MRI. Diffusion MRI is a non-invasive technique sensitive to the random motion of water molecules within tissue, and thus indirectly to the tissue microstructure. In the white matter, diffusion is largest along the principal direction of white matter fibre bundles, and thus is anisotropic. For this reason, it is typically estimated using diffusion tensor (DT) MRI,^41^ which yields parameters such as the mean diffusivity (MD, a directionally averaged measure of the magnitude of diffusion) and fractional anisotropy (FA, which quantifies the degree of directionality). Both indices are known to be altered within MS lesions and in the normal appearing brain tissue of people living with MS.^42^ One of the limitations of the DT model is that it assumes a single water compartment within each voxel, without separating intracellular and extracellular contributions. Therefore, more complex models of diffusion MRI have been proposed. Among these, neurite orientation dispersion and density imaging (NODDI), has gained popularity as it is compatible with clinically feasible scan times.^43^ NODDI allows changes to neurite density (NDI) and orientation dispersion (ODI) to be decoupled, thus providing more specific information on axonal damage than the tensor-derived FA. Demyelination and inflammation can be quantified using quantitative magnetisation transfer (qMT), a technique that indirectly probes macromolecules such as proteins and lipids.^44^ This technique provides the macromolecular pool fraction (F), a validated index of myelination^45^ and the forward exchange rate (*k_f_*), which has been shown to be sensitive to inflammation.^46^

The aim of this study was to investigate the roles of interoception and metacognition in MS fatigue, and their relationship with microstructural tissue damage assessed using quantitative MRI, with the view of identifying potential treatment targets and strategies.

## Materials and methods

### Hypotheses and power calculations

We formulated three alternative hypotheses:

Hypothesis 1 (Interoceptive accuracy): The odds of having high levels of cognitive fatigue differs between MS patients with low and high interoceptive accuracy, whilst interoceptive insight and general metacognitive abilities are not related to fatigue.
Hypothesis 2 (Interoceptive insight): The odds of having cognitive fatigue differ between MS patients with low and high interoceptive insight (the metacognitive aspect of interoception), whilst interoceptive accuracy and other metacognitive abilities are not related to fatigue.
Hypothesis 3 (General metacognition): The odds of having cognitive fatigue differs between MS patients with low and high general metacognitive abilities, including interoceptive insight (the metacognitive aspect of interoception), whilst interoceptive accuracy is not related to fatigue.

In addition, we investigated whether structural white matter damage, assessed using DT MRI, NODDI and quantitative MT, underpins and modulates the relationship between interoception and fatigue through mechanisms of disconnection.

For power calculations, we used G*Power. We aimed to detect a 15% difference in heartbeat discrimination between MS patients with high and low fatigue. With an assumed accuracy score of 55% (SD = 21%) in the low fatigue group, we calculated a sample size of 30 patients per group, which was adjusted to 36, to account for a potential dropout rate of 15%.

This sample size aligns with previous studies on interoceptive ability in similar clinical populations.^47^ Of note, this sample size is consistent with that estimated by Rouault *et al*. using sensitivity analysis.^37^

### Participants and study design

Seventy-one patients with relapsing-remitting MS were recruited from the MS clinic of Brighton and Sussex Universities Hospitals Trust, UK, between April 2017 and May 2018. At recruitment, exclusion criteria for patients were history of other neurological diseases, or the presence of psychiatric and other clinical conditions. In order to rule out potential secondary causes of fatigue, the following criteria were also applied. The depression sub-scale of the hospital anxiety and depression scale (HADS), and the Epworth sleepiness scale (ESS) were used to exclude participants with evidence of depression and sleep disorders at the suggested cut-offs of 11 and 10, respectively.^48,49^ Anxiety was measured using the HADS, but it was not used as an exclusion criterion as it is not an obvious confound for fatigue. Participants with sleep disturbances, on treatment with hypnotics within the last 4 weeks prior enrolment, on recreational drugs, or with known alcohol abuse were excluded. Major abnormalities, such as anaemia, ongoing infections, thyroid dysfunction and vitamin deficiencies, were excluded based on the blood tests performed for clinical purposes. The brief international cognitive assessment for MS^50^ was used to screen for cognitive impairment. Quality-of-life was assessed using the functional assessment in multiple sclerosis (FAMS)^51^ and the EuroQol five dimensions questionnaire with five-level scale (EQ-5D-5L).^52^ Ethical approval was obtained from the London-Surrey Borders Research Ethics Committee (reference = 17/LO/0081). Written informed consent was obtained from all participants according to the declaration of Helsinki. This cohort partially overlaps with those included in three other papers.^21,53,54^

Fatigue was assessed using the modified fatigue impact scale (MFIS).^1^ The total MFIS score (MFIS-Tot; ranging 0–84) is the sum of the cognitive (MFIS-Cog), physical (MFIS-Phys) and psychosocial (MFIS-Soc) sub-scales. In this paper, we restrict our analysis to MFIS-Cog.

When possible, experimental procedures were scheduled at the same time in the afternoon (1–4 p.m.). However, this was not feasible for all participants.

### Interoceptive tasks

We focused on the cardiac axis, and followed the methods described in Garfinkel *et al*.,^35^ which have previously been used in clinical cohorts. In brief, patients performed two separate tasks: the heartbeat tracking task (HTT), and the heartbeat discrimination task (HDT). In the HTT, patients are instructed to silently count each heartbeat they feel during six time-windows of length varying between 25 and 50 s, spaced by 5 s, randomly ordered. The reported count (*nbeats*_reported_) is compared against the actual count (*nbeats*_real_) obtained using a pulse oximeter attached to the index finger. In the HDT, a series of 10 auditory tones is presented to the participant. They need to judge if the tones are synchronous or asynchronous with their heartbeat. Adjusting for the average delay required for the pressure wave to reach the finger after the R-wave, tones are presented at 250 ms (synchronous) or 550 ms (asynchronous) after the R-wave, which correspond to maximum and minimum synchronicity judgements, respectively.^55^ For both tasks, participants are asked to complete a visual analogue scale (VAS), rating their confidence that they gave the correct answer for each trial on a scale from 0 (total guess) to 10 (complete confidence).

For HTT, ‘interoceptive accuracy’ (*I*_acc_) was defined as


(1) $$\begin{array}{rl} I_{\mathrm{acc}}= & 1-(nbeats_{\mathrm{real}}-\;nbeats_{\mathrm{reported}})/(nbeats_{\mathrm{real}}\\ & +\;nbeats_{\mathrm{reported}})/2 \end{array}$$

for each trial and averaged over 26 trials. Interoceptive insight was measured as the within participant Pearson correlation coefficient (*r*) between confidence and accuracy.

For HDT, interoceptive accuracy was calculated dividing the number of correct trials by the number of total trials (correct trials/total trials), while interoceptive insight is calculated according as the area under the curve on a receiver operating characteristic curve using the trial-by-trial correspondence between accuracy (correct synchronous/asynchronous) and confidence assessed using the score on the trial-by-trial VAS.

### Metacognitive tasks

Participants engaged in two metacognitive tasks,^56^ targeting visual perception and memory. They were required to make two-alternative judgements regarding their perceived or memorized stimuli, followed by providing a confidence rating for each decision.

For the visual task, each one of 200 trials (8 blocks of 25 trials each) featured two white circles on a black background, with a variable number of dots (1 to 100) displayed for 0.7 s. Participants were tasked with determining which circle contained more dots. The difficulty level was individually adjusted using a one-up two-down staircase procedure,^56,57^ to maintain a consistent level of difficulty among participants.

In the memory task,^58^ participants were instructed to memorize as many as possible of 50 English words presented on the screen within time intervals of 0.5, 1 or 1.5 min. Subsequently, they underwent a series of two-alternative forced choice judgments, selecting the word they remembered seeing from a list of paired words. Each participant completed a total of 200 memory trials (4 blocks, with 50 trials per block). After each trial in both tasks, participants are presented with a sliding scale (from 1 to 6) to indicate their confidence level in their decision.

Following each trial in both tasks, participants utilized a sliding scale (ranging from 1 to 6) to express their confidence level in their decision. Metacognitive task performance was assessed based on the percentage of correct responses. For the visual perception task, the difficulty threshold was determined as the mean number of dots added or subtracted to the target stimulus through the staircase procedure. Two sensitivity metrics were derived from behavioural data: *d*′, measuring the ability to distinguish stimulus alternatives, and *meta-d*′,^56^ assessing the ability to discriminate correct from incorrect judgements. Metacognitive efficacy, representing the disjunction between objective task performance and subjective confidence, was computed as (*meta-d*′− *d*′) for both metacognitive tasks and subsequently employed in further analyses.

### Magnetic resonance imaging (MRI)

MRI data were acquired on a 1.5T Siemens Magnetom Avanto scanner (Siemens Healthineers, Erlangen, Germany) at the Clinical Imaging Sciences Centre of the University of Sussex, UK. The examination included: (i) a volumetric T1-weighted MPRAGE; (ii) a two-shell diffusion-weighted pulsed-gradient spin-echo EPI; (iii) a qMT scan, based on 3D true fast imaging with steady-state precession; (iv) a T1-mapping sequence, using three 3D fast low-angle shot (FLASH) volumes. Clinical sequences included 2D-dual-echo turbo-spin-echo and 2D-Fast fluid-attenuated inversion recovery. The parameters of the sequences are detailed in the Supplementary materials. Resting-state functional-MRI data were also collected and described in detail elsewhere.^21,53^ In total, the MRI session lasted 45 min.

### Image analysis

The diffusion MRI data were corrected for susceptibility distortions, followed by correction for involuntary movement and eddy current induced distortion using the FSL tools.^59^ The *b*-matrices were rotated to compensate for errors.^37^ FA parameter maps were generated by applying a DT model to each voxel within the corrected data using FSL ‘dtifit’ software. Subject-specific FA maps were then processed using the tract-based spatial statistics (TBSS) pipeline^60^ (https://fsl.fmrib.ox.ac.uk/fsl/fslwiki/TBSS/UserGuide). The corrected data were also analysed using the NODDI fitting algorithm implemented in Matlab and distributed by the developers (http://www.nitrc.org/projects/noddi_toolbox).

For the qMT analysis, we followed the same methods as Harrison *et al*.,^46^ which is based on the balanced steady state free precession qMT model proposed by Gloor *et al*.^61^ The MPRAGE was segmented into tissue classes using statistical parametric mapping (SPM; version 12; Wellcome Trust Centre for Neuroimaging, University College London, UK; http://www.fil.ion.ucl.ac.uk/spm); the white and grey matter segments were then combined to yield a parenchymal mask. The true FISP and the 3D FLASH images were realigned to subject-specific MPRAGE space using SPM12. A T1 map was calculated for all data sets by fitting the theoretical spoiled gradient-echo signal as a function of the flip angle to the signal measured by the 3D FLASH. The qMT parameters F (an index of myelination) and *k_f_* (an index of inflammation) are then calculated by performing a voxel-wise non-linear least-squares fitting (Levenberg–Marquardt method) to a binary spin bath model for balanced steady-state free precession.^61^ The maps were further co-registered with FA, in order to further apply TBSS analysis.

The TBSS analysis was conducted using TBSS^60^ in FSL on the FA maps, using the recommended settings. The non-FA parameter maps from NODDI and MT were skeletonised in the same way, utilising the script *tbss_non_FA*, from FSL.

### Statistical analysis

The statistical analysis of the demographic, clinical and behavioural data was performed in R software, version 4.0.0 (R Foundation for Statistical Computing, Vienna, Austria) and JASP version 0.13.1.0 (University of Amsterdam, Netherlands). The normality of the variable distribution was explored with Shapiro–Wilk and Q–Q (quantile–quantile) plot. The *χ*^2^ test was used to assess the association between two categorical variables. For continuous variables with Gaussian distribution, the homoscedasticity was evaluated by Fisher-F test and a two sample *t*-test was used to identify statistically significant differences in distributions between studied groups. Student’s *t*-test (equal variance) or Welch (unequal variance) tests were used to compare differences in mean values between highly fatigued and mildly fatigued MS patients. Mann–Whitney U-test was used for non-parametric distributions. All types of statistical tests were two-tailed. A test result was considered significant for *P*-values < 0.05. The associations between studied interoception and metacognition factors and the odds of high or low cognitive fatigue were tested by unconditional binomial logistic regression. First, univariate regression analysis was performed. Then, the relevant predictors (with unadjusted *P*-value ≤ 0.25^62^) and the most clinically relevant covariates were included in the multivariate regression analysis. Full additive multivariate logistic regression models were tested, with the aim to evaluate whether the interoception (accuracy and insight, for both tasks) and metacognition factors (efficiency for both tasks) were associated with the odds of high cognitive fatigue, adjusting for age, sex, disease duration, expanded disability status scale (EDSS, a measure of disability), anxiety and depression. The assumptions of linearity between logit transformation of high cognitive fatigue and continuous covariates included in the logistic regression models were verified by using a smooth function from the *mgcv* R package.^63^ The predictors which did not respect the linearity condition were dichotomized and then included in the full model.

For imaging data, the 4D skeletonized parameter files were entered into voxel-wise statistical analysis using FSL *randomise_parallel*, applying the 2D threshold-free cluster enhancement (TFCE) correction for multiple comparisons.^64^ A general linear univariate model^65^ was used, setting the significance level after TFCE correction at *P* < 0.05. Correlation tests were run between MRI parameters and cognitive fatigue.

*Post hoc*, the effect on MRI parameters of the interaction between cognitive fatigue and interoceptive/metacognitive measures predictive of fatigue was tested. The relevant behavioural variable was categorized into two levels, using the median value as a threshold. The analysis was performed in *randomise_parallel,* with the behavioural variable, cognitive fatigue and their interaction as factors, and skeletonized images as the dependent variable. The same correction for multiple comparisons and criteria for significance used for the correlation analysis were applied. For the purpose of interpreting the interaction effects, we extracted and plotted the mean parameter values for the voxels that resulted significant.

## Results

### Sociodemographic and clinical information

All patients completed the questionnaires and the interoceptive tasks, 69 completed the MRI session, and 67 at least one of the metacognitive tasks. Sixty-six completed both metacognitive tasks. As the protocol required a maximum of 2-weeks between screening and experimental procedures, in two cases, it was not possible to book the MRI scanner within this interval. In all the other cases, missing data were due to the participant’s availability to complete the session, and not to their ability to tolerate the procedures.

Fifty-four patients were under disease-modifying treatment (DMTs) (Alemtuzumab: *N* = 16, Dimetylfumarate: *N* = 11, Natalizumab: *N* = 7, Teriflunomide: *N* = 4, Glatiramer Acetate: *N* = 7, Fingolimod: *N* = 6, Beta-interferons: *N* = 3). As there is no generally accepted cut-off for MFIS-Cog, the median value of 16 was used. This value is higher than the mean value observed in larger cohorts,^66^ and therefore patients with MFIS-Cog ≥ 16 were allocated to the high cognitive fatigue group (*N* = 38), leaving 33 (MFIS-Cog < 16) in the mild cognitive fatigue group. The high cognitive fatigue and mild cognitive fatigue groups were balanced in terms of age (*P* = 0.764), education years (*P* = 0.977), and sleepiness (ESS *P* = 0.665). However, they differed in disability (the high cognitive fatigue group had significantly higher EDSS scores, *P* < 0.001), anxiety (the high cognitive fatigue group had significantly higher HADS-A, *P* = 0.047) and depression (the high cognitive fatigue group had significantly higher HADS-D, *P* = 0.015). Table 1 summarises demographic and clinical characteristics of the two groups. DMTs were classified as either moderate efficacy (beta-interferon, Glatimer acetate, dimethyl-fumarate and teriflunomide) or high efficacy (fingolimod, natalizumab and alemtuzumab), and the groups were balanced in terms DMT repartition when using this classification (*χ²* = 0.1, *P* = 0.950).

**Table 1:** Demographic and clinical data of the participants

	Fatigued (*N* = 38)	Non-fatigued (*N* = 33)	*P*-value
M/F	16/22	22/11	0.448^a^
Median age (IQR) [years]	43.5 (38.3–50)	45.0 (39–49)	0.764^b^
Median education (IQR) [years]	16 (12–18.75)	17 (14–18)	0.977^b^
Median EDSS (IQR)	2.5 (1.5–4)	1.5 (1–2)	<0.001^b^
Median DD (IQR) [years]	6 (3–10.75)	17 (14–18)	0.092^b^
Median SDMT (IQR)	47.5 (42–52.75)	50 (47–56)	0.087^b^
Median BVMTR (IQR)	26.5 (19.25–31.5)	27 (24–31)	0.182^b^
Mean CVLT (SD)	57 (9.97)	55.88 (12.03)	0.669
Mean ESS (SD)	4.68 (2.98)	4.39 (2.6)	0.665
Median HADS-D (IQR)	2.5 (1–4)	1 (1–2)	0.015^b^
Mean HADS-A (SD)	5.24 (2.88)	3.85 (2.88)	0.047
Median FAMS (IQR)	119 (97–130)	141 (133–158)	<0.001^b^
Median EQ-5D-5L (IQR)	8 (6–10)	6 (5–8)	0.001^b^
DMT (D0/D1/D2)	9/14/15	8/11/14	0.95^a^

Abbreviations: M, male; F, female; IQR, inter-quartile range; SD, standard deviation; EDSS, expanded disability status score; DD, disease duration; SDMT, symbol digit modalities test; BVMTR, brief visuospatial memory test revised; CVLT, California verbal learning test II; ESS, Epworth sleepiness scale; HADS-A, anxiety sub-scale of the hospital anxiety and depression scale; HADS-D, depression sub-scale of the hospital anxiety and depression scale; FAMS, functional assessment in multiple sclerosis; EQ-5D-5L, EuroQol five dimensions questionnaire with five-level scale; DMT, disease-modifying treatment (D0 = no treatment; D1 = moderate efficacy treatment; D2 = high efficacy treatment). *P*-values in bold indicate significant between-group differences.

Statistical comparisons were performed using an independent sample *t*-test, unless otherwise specified.

^a^The *χ*2 test.

^b^Mann–Whitney U-test.

No significant difference in cognitive function was found between cognitively highly fatigued and mildly fatigued MS patients (California verbal learning test: *P* = 0.7; symbol digit modality test: *P* = 0.09; brief visuospatial memory test-revisited: *P* = 0.18). However, the median quality of life was significantly lower in the high cognitive fatigued group, when measured with the FAMS (*P* < 0.001) and EQ-5D-5L questionnaires (*P* = 0.001).

### Interoception and metacognition

Table 2 summarises the results of the logistic regression models, investigating the odds of differences in cognitive fatigue associated with the interoceptive and metacognitive scores, for both unadjusted models and models adjusted for age, sex, disease duration from diagnosis, EDSS level, anxiety and depression. Tracking (HTT) insight was the only regressor of interest found to be statistically significant as predictive factor for high cognitive fatigue, both in the univariate (OR = 0.29, 95% CI: [0.11, 0.76], *P* = 0.014) and multivariate model (OR = 0.26, 95% CI: [0.07, 0.84], *P* = 0.029).

**Table 2:** Logistic regression models for the odds of higher cognitive fatigue in patients with MS being associated with heartbeat tracking, heartbeat discrimination and metacognition variables

Cognitive fatigue	Estimated regression coefficients (SE)	OR [95% CI]	*P*-value	Estimated regression coefficients (SE)	OR [95% CI]	*P*-value
Model 1	Model 1 unadjusted			Model 1 adjusted		
Tracking insight^a^	−1.23 (0.50)	0.29 [0.11, 0.76]	0.014	−1.36 (0.62)	0.26 [0.07, 0.84]	**0**.**029**
Tracking accuracy^b^	0.39 (0.49)	1.48 [0.58, 3.83]	0.411	0.44 (0.59)	1.55 [0.49, 5.03]	0.454
Model 2	Model 2 unadjusted			Model 2 adjusted		
Discrimination insight^c^	−0.52 (0.48)	0.596 [0.23, 1.52]	0.282	−0.65 (0.61)	0.52 [0.15, 1.67]	0.285
Discrimination accuracy^d^	0.72 (0.49)	2.06 [0.8, 5.42]	0.138	1.01 (0.61)	2.76 [0.86, 9.71]	0.098
Model 3	Model 3 unadjusted			Model 3 adjusted		
Visual perception metacognition^e^	−0.41 (0.48)	0.66 [0.26, 1.69]	0.392	−0.62 (0.60)	0.54 [0.16, 1.70]	0.297
Memory metacognition^f^	0.15 (0.48)	1.16 [0.46, 2.98]	0.753	0.28 (0.59)	1.32 [0.41, 4.28]	0.641

Outcome variable: Cognitive fatigue defined as a dichotomized variable (higher/lower status where higher was defined as cMFIS ≥ 16 points). Unadjusted models represent univariate logistic regression models; adjusted models represent the multivariate logistic regression models adjusted for age, sex, disease duration from diagnosis, EDSS level, anxiety and depression. All tested explanatory variables were introduced in the regression models as dichotomized variables (higher/lower status); the cut-off point for higher status was the median value of the studied predictor.

^a^ ≥ 0.195 versus <0.195.

^b^≥ 0.668 versus <0.668.

^c^ ≥ 0.513 versus <0.513.

^d^ ≥ 0.5 versus <0.5.

^e^≥ 0.324 versus <0.324.

^f^≥ 0.1935 versus <0.1935.

*P*-values in bold indicate statistical significance.

### Correlations between MRI and behavioural variables

**Figure 1:**
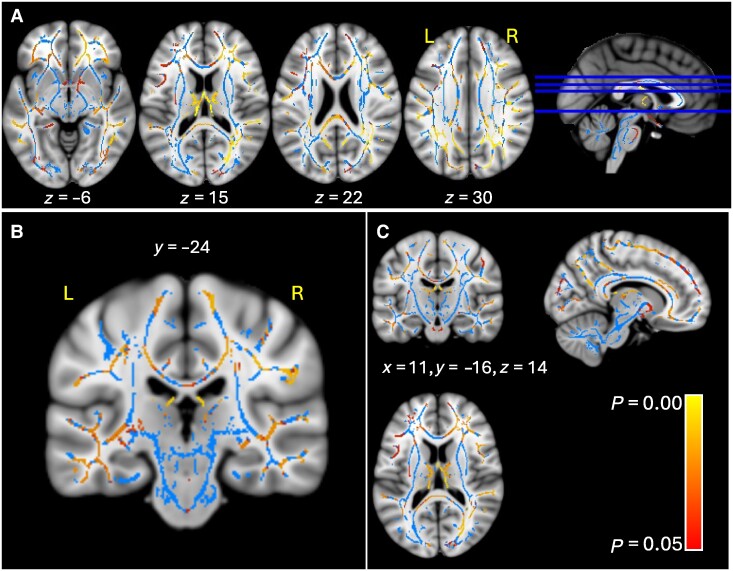
**Inverse correlation between cognitive fatigue and diffusion parameter FA.** Results include NAWM and MS lesions. Area of significant association is shown using a red–yellow scale (corresponding to *P* values ranging from 0.000 to 0.05), overlaid on top of the FSL MNI T1-weighted template, and the white matter skeleton (in light blue). Axial sections are shown in panel (**A**), a coronal slice in panel (**B**) and orthogonal sections are shown in panel (**C**). MNI coordinates are shown for reference.

**Figure 2:**
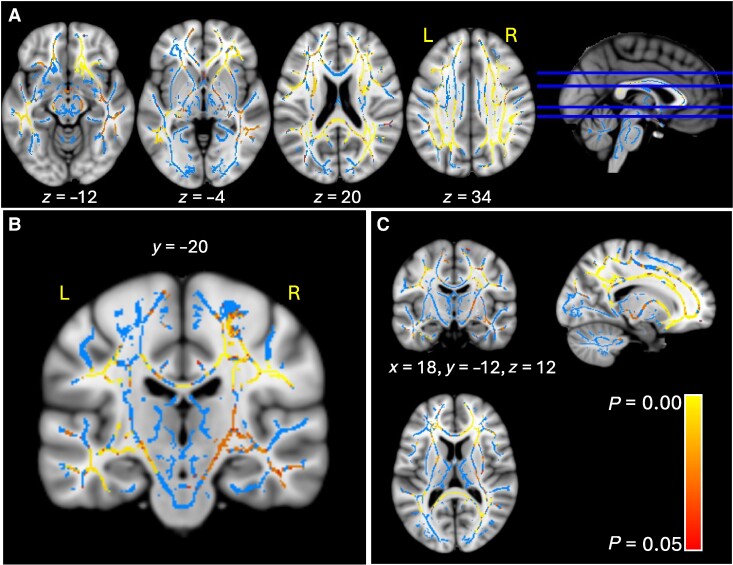
**Inverse correlation between cognitive fatigue and NDI results include NAWM and MS lesions.** Area of significant association is shown using a red-yellow scale (corresponding to *P* values ranging from 0.000 to 0.05), overlaid on top of the FSL MNI T1-weighted template, and the white matter skeleton (in light blue). Axial sections are shown in panel (**A**), a coronal slice in panel (**B**) and orthogonal sections are shown in panel (**C**). MNI coordinates are shown for reference.

TBSS analysis revealed an inverse correlation between cognitive fatigue and both, FA (Fig. 1) and NODDI NDI (Fig. 2) in a widespread bilateral white matter network. Both FA and NDI were inversely correlated with MFIS-Cog in the superior longitudinal fasciculus, medial longitudinal fasciculus, inferior longitudinal fasciculus, arcuate and uncinated fasciculi, inferior fronto-occipital fasciculus (particularly in the right hemisphere), external capsule, cingulum, the cingulum connections with the parietal lobe, corpus callosum (body and splenium), callosal radiations, forceps major, forceps minor, fornix, hippocampal commissure, U fibres (mainly frontal and parietal) and orbito-medial prefrontal connecting fibres. The correlation with FA was no longer significant when adjusting for depression, disability and disease duration. These covariates were chosen as they may affect the feeling of fatigue.

By contrast, the correlation with NDI remains significant in right orbito-medial prefrontal connecting fibres, with involvement of parts of the right thalamic radiations (anterior limb) and right U fibres (frontal, parietal) (alpha ≥ 0.95, *P* ≤ 0.05). No significant correlation was found between cognitive fatigue and the other NODDI parameters (iso, ODI), or any of the qMT parameters.

### Interaction between cognitive fatigue and heartbeat tracking insight and effects on MRI parameters

**Figure 3:**
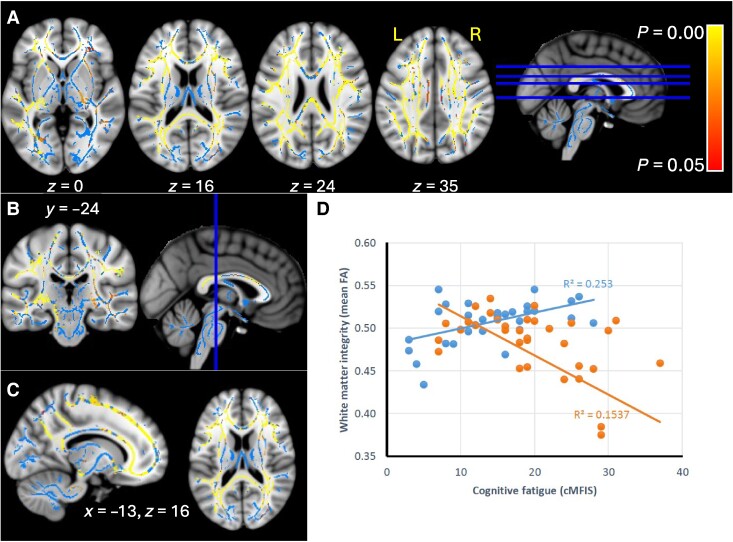
**Significant interaction effect of cognitive fatigue and interoceptive heartbeat tracking insight, on TBSS FA*—*in red–yellow**. Results for NAWM and MS lesions, shown for selected sections, overlaid on skeleton (blue) and MNI T1-weighted template (**A–C**). Scatter plot for the interaction effects of cognitive fatigue and interoceptive tracking insight (high = blue; low = orange) on FA, *N* = 69 (**D**).

The interaction between cognitive fatigue and interoceptive tracking insight (split into two levels) was significant (*P* ≤ 0.001) for FA within a bilateral widespread network, including superior longitudinal fasciculus, medial longitudinal fasciculus, inferior longitudinal fasciculus, arcuate fasciculus, uncinate fasciculus, inferior fronto-occipital fasciculus, external capsule, cingulum, cingulum connections with the parietal lobe, corpus callosum (rostrum, genu, body, splenium), callosal radiations, forceps major, forceps minor, fornix, hippocampal commissure, posterior thalamic radiations and genu of white capsule, optic radiations, U fibres (frontal, parietal and temporal) and orbito-medial prefrontal connecting fibres (Fig. 3A–C).

To further interpret this result, the average cluster’s FA against cognitive fatigue was plotted in Fig. 3D. The plot shows a positive association between fatigue and FA in patients with high tracking insight, and a negative association in those with low tracking insight. When controlling for depression, disability and disease duration, the interaction remains significant at *P* ≤ 0.05, although the extent of the significant clusters is reduced (Supplementary Fig. 1). Very similar results were found for NODDI NDI (Supplementary Fig. 2). By contrast, the interaction was not significant for ISO and ODI (alpha < 0.98, *P* > 0.05), thus suggesting that FA findings are primarily explained by microstructural changes to the white matter tracts rather than changes in the distribution of fibre orientations.

**Figure 4:**
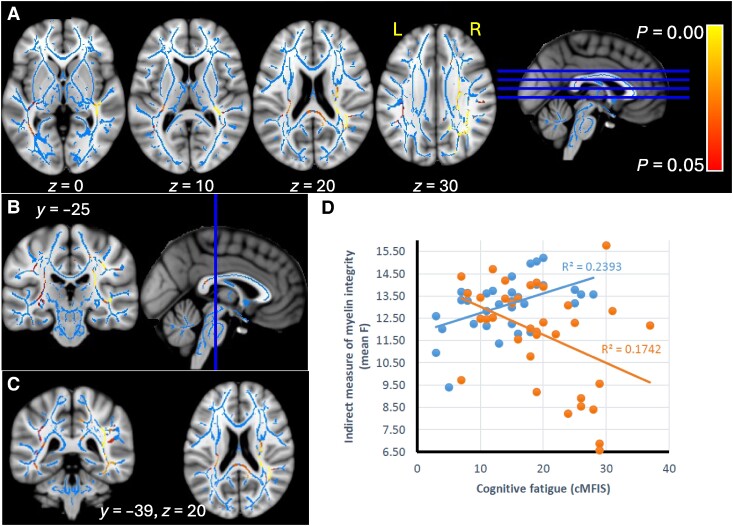
**Significant interaction effect of cognitive fatigue and interoceptive heartbeat tracking insight, on TBSS MT parameter F—in red–yellow.** Results for NAWM and MS lesions, shown for selected sections, overlaid on skeleton (blue), and MNI T1-weighted template (**A–C**). Scatter plot for the interaction effects of cognitive fatigue and interoceptive tracking insight (high = blue; low = orange) on F, *N* = 69 (**D**).

The interaction between MFIS-Cog and interoceptive tracking insight was significant also for the qMT-derived indices F and *k_f_*. The former result was found at the level of the right superior longitudinal fasciculus, right medial longitudinal fasciculus, right inferior longitudinal fasciculus, arcuate fasciculus, right cingulum bundle, right cingulum connections with the parietal lobe, corpus callosum (splenium), forceps minor, posterior thalamic radiations and right parietal U fibres (Fig. 4A–C).

**Figure 5:**
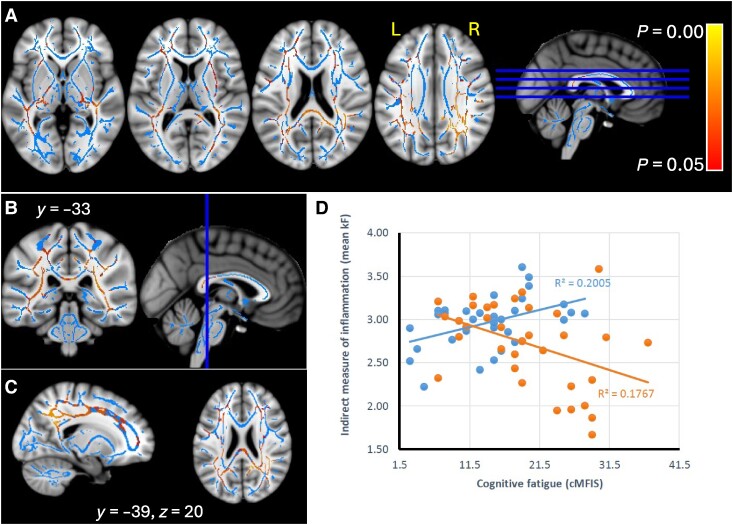
**Significant interaction effect of cognitive fatigue and interoceptive heartbeat tracking insight, on TBSS MT parameter *k_f_*—in red–yellow**. Results for NAWM and MS lesions, shown for selected sections, overlaid on skeleton (blue), and MNI T1-weighted template (**A–C**). Scatter plot for the interaction effects of cognitive fatigue and interoceptive tracking insight (high = blue; low = orange) on *k_f_*, *N* = 69 (**D**).

The effect on *k_f_* was significant in the superior longitudinal fasciculus, medial longitudinal fasciculus, arcuate fasciculus (more on the left), left uncinate fasciculus, external capsule, right cingulum bundle, cingulum connections with the parietal lobe (more on the right), corpus callosum (splenium), callosal radiations, forceps major, thalamic radiations (posterior limb) and optic radiations and U fibres (frontal and parietal) (Fig. 5A).

The data plot in Figs 4D and 5D indicate that in people with low tracking insight, the lower the qMT parameter, the higher cognitive fatigue is, while the reverse is true for people with high tracking insight. However, when controlling for depression, disability and disease duration, the interaction is no longer significant for either qMT variable.

## Discussion

This paper explores the underpinnings of cognitive fatigue in MS by combining behavioural and MRI analyses. We explored three alternative hypotheses, investigating whether interoceptive accuracy, interoceptive insight or general metacognition more significantly modulate cognitive fatigue. Our findings indicate that participants with low interoceptive insight (i.e. the metacognitive aspect of interoception), as assessed by the HTT, have higher odds of experiencing elevated cognitive fatigue. No association was found between cognitive fatigue and impaired global metacognitive abilities.

Interoceptive insight is regarded as a sub-domain of metacognition. A controversial topic is whether metacognition draws from a global resource applicable across various tasks or is task-specific.^67^ The prevailing consensus acknowledges the coexistence of both domain-specific and domain-general metacognition representations, with a potential gradient in which some tasks (such as different types of perceptual judgment) are more likely to rely on shared circuitry for metacognitive evaluation than others.^67^ Given this background, it is reasonable to explore whether a general metacognitive deficit, rather than a specific interoceptive insight deficit, could be linked to fatigue in MS. In this study, we used well established and widely adopted tasks to measure visual perception and memory metacognition, and we found that the odds of having cognitive fatigue do not significantly differ between people with either low and high visual perception or memory metacognitive abilities, thus suggesting that for interoception related to MS fatigue, the gradient of sub-domain specificity is high. Importantly, our behavioural results replicate those obtained by Rouault *et al*.^37^ in an independently conducted study, based on self-reported assessment of metacognitive insight. This consistency strengthens the reliability of our results.

We used two separate interoceptive tasks, and we only found significant results for interoceptive insight when using the HTT. This discrepancy is not unexpected, as a recent meta-analysis^68^ found no significant correlation between HTT and HDT insight (0.8% variance shared). This lack of correlation is partially explained by the different domains the two tasks rely on: in HTT working memory and sustained attention is needed, whereas in HDT multisensory integration of exteroceptive and interoceptive stimuli is required. However, it is important to consider some of the potential sources of bias that might have affected our results. First, we cannot exclude the possibility that participants might use non-interoceptive strategies, for example beliefs concerning the heart rate^69-72^ and time estimation abilities^73^ in the HTT.^74,75^ These are well acknowledged shortcomings of the HTT. This task has the advantage of being relatively short to complete, and easily accessible to patients with fatigue. On the other hand, it has been argued that this measure serves as a poor test of interoceptive accuracy as strategies not dependent upon the detection of internal bodily signals can guide better performance accuracy.^71,76^ Our study attempted to mitigate this criticism by providing a trial-by-trial VAS, which explicitly requests that participants report whether their heartbeat estimate derives from a ‘total guess (no heartbeat awareness)’ to ‘complete confidence (full perception of heartbeat)’. As insight was measured as the correlation between confidence and accuracy, it is worth noting that we only included six trials for this task, so correlations mapping confidence to accuracy are unstable, and require further replication with more trials. By contrast, while the HDT cannot be completed by higher order knowledge of heartrate (as tones are presented at the same temporal frequency irrespective of whether they are in synch or out of synch with heartbeats), this task assumes that all participants ‘feel’ their heart at a certain point in the cardiac cycle. Some tasks, such as multiple interval tasks based on psychophysical methods^69^ vary the tones in relation to R wave at 100 ms intervals, allowing for individual differences in when (i.e. at what point in the cardiac cycle) individuals might reliably detect their heart beating. While this procedure accommodates individual differences in this parameter, it is also much longer, rendering this a less suitable task for people with fatigue. Instead, we chose two points in the cardiac cycle that are maximally ‘distinct’, with the ‘synchronous’ tone occurring at the point in the cardiac cycle where the majority of participants are likely to sense their heartbeat.^55^ We administered only 26 trials, which is less than the recommended amount of 40–60.^77^

Interestingly, in this study interoceptive accuracy was not found to be predictive *per se*. Rather, it was the confidence measure ‘in relation to accuracy’ that was the significant predictor in our model. This result suggests that MS cognitive fatigue does not arise through faulty communication from the body to the brain, but rather the faulty processing occurs at brain level. This observation aligns with the expectation that damage to both white and grey matter may underlie the observed interoceptive deficit in MS. Our neuroimaging analysis further supports this notion, indicating that the interaction between cognitive fatigue and interoception insight manifests in the microstructure of white matter. However, it is noteworthy that our results, particularly the lack of significant findings regarding interoceptive accuracy, diverge in part from a prior study.^78^ In that study, the association was deemed significant, but it focused on total fatigue, encompassing cognitive, physical and social aspects.

Our study highlights a widespread structural network within the white matter skeleton, specifically linked to cognitive fatigue in MS. Notably, prior investigations into the association between DT MRI metrics and general fatigue, as opposed to cognitive fatigue, have generated relatively inconsistent results.^39,79,80^ Our findings point to reduced FA, reflecting microscopic damage to the white matter, in pathways connecting key nodes of the interoceptive network and the reward system (e.g. superior longitudinal fasciculus, uncinate fasciculus, cingulum and thalamic radiations). This reduction in FA tends to be associated with increased cognitive fatigue. In addition to DT MRI, we used a multimodal neuroimaging approach, including NODDI and qMT parameters, which allow us to characterize the observed tissue changes with improved specificity. The general overlap between FA and NDI results suggests that the observed correlations are mainly driven by microscopic effects (axonal density) rather than the macroscopic effects (ODI), which are intertwined in FA.

The interaction analysis further validates that the association between cognitive fatigue and axonal damage is driven specifically by people with low interoceptive insight. This observation underscores the nuanced interplay between structural changes in white matter and cognitive fatigue in the MS brain, supporting the hypothesis that disconnection within the relevant functional circuits might subtend the link between interoceptive insight and fatigue.

It is noteworthy that we observed no direct association between qMT indices, mostly linked to demyelination and inflammation, and fatigue. To our knowledge, there is only one study by Andreasen *et al*.^81^ that delved into white matter changes in MS fatigue using magnetisation transfer. Consistent with our results, this study did not identify magnetisation transfer ratio differences in the normal appearing white matter (NAWM) between individuals with high and mild MS fatigue. Another magnetisation transfer study, exploring grey matter in 14 fatigued and 14 non-fatigued MS patients,^79^ also yielded non-significant results.

Given the methodological disparities—utilizing magnetisation transfer ratio versus qMT—and the modest sample sizes (both studies relying on fewer than 20 participants per group), comparing our results with theirs is not straightforward. Turning to our present findings, the absence of a significant correlation between qMT parameters and cognitive fatigue suggests that axonal involvement is the primary mechanism modulating this symptom in MS.

These outcomes align with the hypothesis that cognitive fatigue in RRMS may not primarily result from inflammation but rather stem from disconnection, directly impacting the white matter pathways that support interoceptive-insight networks. This emphasizes the intricate relationship between structural changes in the brain and cognitive fatigue in the context of MS.

In addition to the potential shortcomings of the HTT, this study suffers from other limitations. We focused on relapsing-remitting MS, and the exclusion of progressive forms might impact generalizability.^82^ Additionally, the lack of correlation between qMT parameters and cognitive fatigue could be influenced by the exclusion of depressed patients, as per the ASE theory.^36^ We focused on the white matter skeleton, without isolating macroscopic lesions from the NAWM. The rationale for this approach was to consider disconnection as the primary mechanism. The existing literature discounts a direct relationship between lesion volume and fatigue. Nevertheless, we cannot exclude that the results might be driven by a handful of patients with larger lesions in eloquent areas. Finally, due to the complexity of our study, we had to limit the number of covariates, and decided to focus on disability, disease duration and depression. However, other clinical variables, such as alexithymia, have been previously found to be associated with fatigue.^83^ We also wish to reiterate that, due to the relative small events per predictor and exploratory nature of the present study regarding the potential factors linked with the higher cognitive fatigue in patients with MS, the results of multivariable models should be regarded as preliminary and interpreted with caution, further studies being required to validate the interoceptive insight (heartbeat tracking insight) measure as independent predictor for higher cognitive fatigue.

In conclusion, our study provides evidence that MS fatigue is partially explained by a deficit in interoceptive metacognition, linking this deficit to axonal damage in specific white matter tracts. This opens avenues for potential interventions, such as training programmes targeting interoceptive metacognition. Metacognition has already been considered as a potential target of interventions in psychiatric disorders including schizophrenia and depression.^84^ While MS fatigue does not seem to correlate with general metacognition, leading to the logical inference that general metacognition training may not be effective, these studies indicate the feasibility of training metacognition in general. A prior attempt at a standardized metacognitive intervention in managing neuropsychological symptoms in MS, though not specifically focused on fatigue, did not yield success.^85^ Although this study was not focused on fatigue *per se*, the Fatigue Scale for Motor and Cognitive Functions, was included among the outcome variables, and showed no significant improvement, following the intervention. Therefore, these preliminary findings suggest that effective training should be directed specifically towards interoceptive metacognition. Intriguingly, Quadt *et al*.^86^ have demonstrated that interoceptive accuracy can also be trained, leading to reduced anxiety in adults with autism spectrum disorder. Taken together, these examples of successful interventions targeting different dimensions of metacognition and interoception raise the possibility of combining the two approaches to develop an interoceptive metacognition training paradigm for MS fatigue. Building on our neuroimaging findings and the conclusion that microstructural damage contributes to impaired interoception insight, we propose that a successful training programme would rely on mechanisms of white matter plasticity. It is conceivable that such an intervention might only be effective for patients with relative brain tissue preservation, suggesting that beyond a certain degree of tissue damage, a full recovery of interoceptive metacognition may not be possible. Therefore, exploring whether there exists a window of opportunity for deploying such a treatment, leveraging on plastic adaptations, remains a crucial avenue for further investigation.

## Conclusions

The behavioural hypotheses tested in this study confirm a specific mechanism of metacognitive interoception associated with fatigue in MS, in line with the dyshomeostatic theory of MS fatigue^18^ and the results of a recent study.^37^ These results informed the MRI analysis, which in turn suggests the involvement of a microstructurally compromised widespread white matter network with MS fatigue, including connections of key interoceptive and reward systems. These data set the foundations for exploring potential treatment options for MS fatigue.

## Supplementary Material

fcae292_Supplementary_Data

## Data Availability

MRI data are available from the corresponding author upon reasonable request, providing signature of an appropriate data transfer agreement. Image analysis was based on open source tools from SPM and FSL.
