# Supplementary material for: Understanding the mechanisms of fatigue in multiple sclerosis: linking interoception, metacognition and white matter dysconnectivity
Source: Brain Commun. 2024 Sep 11;6(5):fcae292. doi: 10.1093/braincomms/fcae292 (PMC11406465; doi:10.1093/braincomms/fcae292)
Supplement: fcae292_Supplementary_Data [file fcae292_supplementary_data.pdf]

Supplementary Material

MRI acquisition parameters

The parameters of the MRI acquisition protocol were as follows:

1) Volumetric T1-weighted MPRAGE (TE=3.57ms; TR=2730ms; TI=100ms; flip-angle=7°; matrix=254x40x192; voxel size=1mm isotropic);
2) two-shell diffusion-weighted pulsed-gradient spin-echo EPI (TE = 95 ms, TR = 4036, b values = 800/2000 s/mm^2, number of diffusion directions = 30/60, FoV = 240 × 240 mm^2, matrix = 96 × 96, slice-thickness = 2.5 mm), with 9 images with no diffusion weighting (b_0). Three of the b_0 volumes were acquired with reversed gradient blips to enable correcting for susceptibility artefacts (Andersson and Sotiropoulos, 2016);
3) qMT scan, based on 3D True Fast Imaging with Steady-state Precession (True FISP; FoV = 240 x 180mm2, Matrix = 256 x 96, slice thickness = 5 mm); 24 volumes were acquired varying either the flip angle (between 5° and 40°) or the repetition time (between 3.66ms and 5.96ms) and the pulse duration (0.2-2.5ms);
4) T1-mapping sequence, using three 3D fast low-angle shot (FLASH) volumes are acquired for T1-mapping, with repetition time 30ms and echo time 5ms. The excitation flip angles are varied between volumes (50°, 150°, 250°). The same field of view, matrix, and number of slices as the True FISP are used.
5) 2D-dual-echo turbo-spin-echo (TSE; TEs = 11/86 ms, TR = 3040 ms, echo-train-length = 6, flip-angle = 150°, FoV = 220 × 192mm2; matrix = 256 × 224; slice-thickness = 5 mm);
6)2D-Fast fluid-attenuated inversion recovery (FLAIR; TE = 87 ms, TR = 8000 ms, TI = 2500 ms, flip-angle = 150°, echo-train-length = 17, same resolution and slice thickness as the dual echo).

Supplementary Figure 1

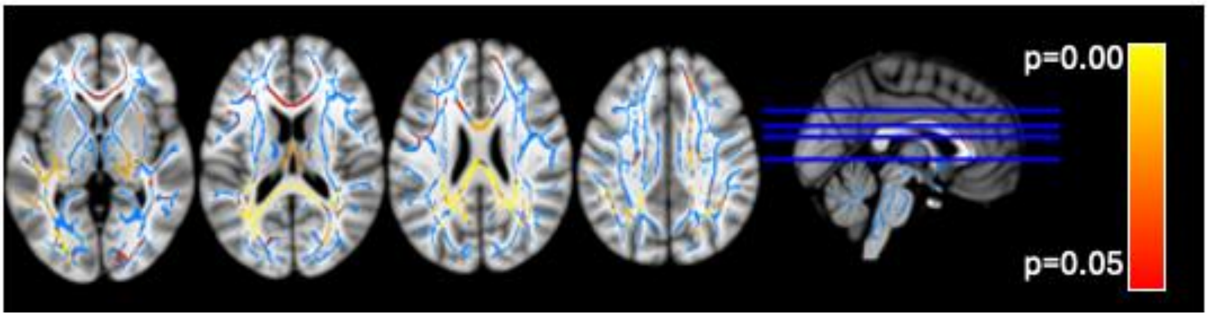

Supplementary Figure 1: Areas of significant interaction of cognitive fatigue and interoceptive heartbeat tracking insight, on TBSS fractional anisotropy (FA) when accounting for disability, depression and disease duration – in red-yellow scale. The analysis was performed using the tool randomise_parallel from FSL, applying the 2D threshold-free cluster enhancement (TFCE) correction for multiple comparisons, and accepting as significant p values of less than 0.05. Interoceptive insight, cognitive fatigue and their interaction were modelled as factors, and skeletonised FA images as the dependent variable. Results for normal appearing white matter and MS lesions, shown for selected sections, overlaid on skeleton (blue), and MNI T1-weighted template.

Supplementary Figure 2

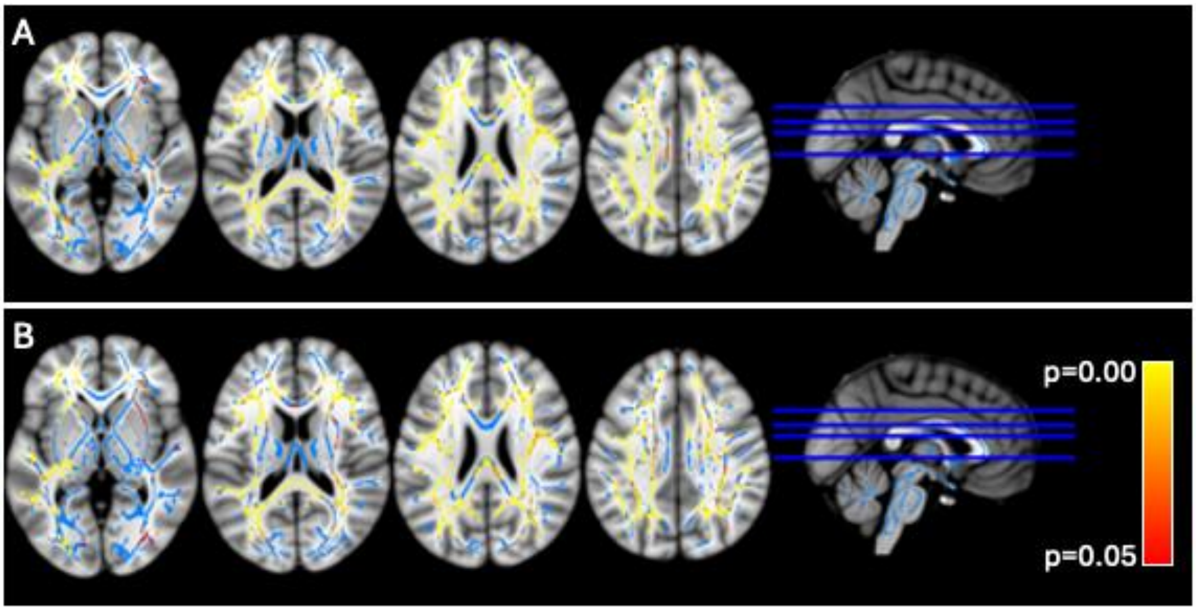

Supplementary Figure 2: Areas of significant interaction of cognitive fatigue and interoceptive heartbeat tracking insight, on TBSS neurite density imaging (NDI) without (A) and with (B) accounting for disability, depression and disease duration. The analysis was performed using the tool randomise_parallel from FSL, applying the 2D threshold-free cluster enhancement (TFCE) correction for multiple comparisons, and accepting as significant p values of less than 0.05. Interoceptive insight, cognitive fatigue and their interaction were modelled as factors, and skeletonised NDI images as the dependent variable. Significant effects are sown in red-yellow scale and overlaid on skeleton (blue), and MNI T1-weighted template. Results include normal appearing white matter and MS lesions.
